# Supplementary material for: Comparative multiomics analysis of cell physiological state after culture in a basket bioreactor
Source: Sci Rep. 2022 Nov 23;12:20161. doi: 10.1038/s41598-022-24687-4 (PMC9686226; doi:10.1038/s41598-022-24687-4)
Supplement: Supplementary file 1 — Supplementary Information 1. [file 41598_2022_24687_MOESM1_ESM.zip › raw data/Transcriptomic raw data/6.Enrichment/2.KEGG/TE1vsTC1/ALL/src/csab04062.html]

csab04062


- csab:103235798

- Down regulated genes

103235789(-1.64077436097819)
- csab:103242198

- Down regulated genes

103242198(-2.54075217125675)
- csab:103242710

- Down regulated genes

103242710(-1.6025804891077)

- csab:103224900

- Down regulated genes

103224900(-1.42998371610984)

Close
